# Treatment comparison of hydroxyurea versus ruxolitinib in essential thrombocythaemia: A matched‐cohort analysis

**DOI:** 10.1002/jha2.954

**Published:** 2024-07-19

**Authors:** Michael R. Grunwald, Ellen K. Ritchie, Elisa Rumi, Albert Assad, J. E. Hamer‐Maansson, Jingbo Yu, Tricia Kalafut, Evan Braunstein, Francesco Passamonti

**Affiliations:** ^1^ Department of Hematologic Oncology and Blood Disorders Levine Cancer Institute Atrium Health Charlotte North Carolina USA; ^2^ Weill Cornell Medical College and New York Presbyterian Hospital New York New York USA; ^3^ Department of Molecular Medicine University of Pavia Pavia Italy; ^4^ Division of Hematology Fondazione IRCCS Policlinico San Matteo Pavia Italy; ^5^ Incyte Corporation Wilmington Delaware USA; ^6^ Università degli Studi di Milano Policlinico di Milano Ospedale Maggiore, Fondazione I.R.C.C.S Ca Granda Milano Italy

**Keywords:** essential thrombocythaemia, hydroxyurea, matched‐cohort analysis, ruxolitinib

## Abstract

Hydroxyurea is the preferred first‐line cytoreductive treatment for high‐risk essential thrombocythaemia (ET), but many patients are intolerant or refractory to hydroxyurea. Ruxolitinib has been shown to improve symptoms in patients with ET. This post hoc analysis compared the clinical outcomes of patients with ET who received hydroxyurea only with those who switched from hydroxyurea to ruxolitinib due to intolerance/resistance to hydroxyurea. Patients with ET refractory/intolerant to hydroxyurea treated with ruxolitinib in a completed phase 2 study (HU‐RUX) were propensity score matched with patients who received hydroxyurea only in an observational study (HU). Changes in leukocyte and platelet counts were reported at 6‐month intervals during the 48‐month follow‐up. Following propensity score matching, 37 patients were included for analysis in each cohort. Mean (standard deviation [SD]) leukocyte and platelet counts at index were higher for HU‐RUX versus HU (leukocyte: 9.3 [5.1] vs. 6.8 [3.1] × 10^9^/L; platelet: 1027.4 [497.8] vs. 513.9 [154.7] × 10^9^/L), both of which decreased significantly from index to 6 months through to 48 months in HU‐RUX (mean [SD] change from index at 6 months—leukocyte: −1.8 [4.6] × 10^9^/L; platelet: −391.7 [472.9] × 10^9^/L; at 48 months—leukocyte: −3.8 [5.3] × 10^9^/L; platelet: −539.0 [521.8] × 10^9^/L), but remained relatively stable in HU (mean [SD] change from index at 6 months—leukocyte: 0 [1.8] × 10^9^/L; platelet: −5.7 [175.3] × 10^9^/L; at 48 months—leukocyte: −0.1 [2.7] × 10^9^/L; platelet: −6.9 [105.1] × 10^9^/L). In conclusion, these results demonstrate that switching from hydroxyurea to ruxolitinib in patients with ET who are intolerant or refractory to hydroxyurea could improve abnormal haematologic values similar to those who receive first‐line hydroxyurea.

## 1 INTRODUCTION

Essential thrombocythaemia (ET) is a myeloproliferative neoplasm characterised by clonal blood cell proliferation and excess platelet production [1]. Patients with ET have an elevated risk of haemorrhagic and thrombotic events (TEs), myelofibrotic or leukaemic transformation, reduced life expectancy and reported lower quality of life compared with matched controls [1].

Hydroxyurea, also known as hydroxycarbamide, is recommended as first‐line cytoreductive therapy in ET [1] and has shown efficacy in lowering platelet counts and reducing the risk of thrombosis and vascular complications in several randomised controlled trials [2, 3]. Additionally, a retrospective cohort study reported that hydroxyurea treatment is associated with a significantly lower risk of death in older patients with ET [4]. However, intolerance and/or resistance to hydroxyurea can occur in up to 25% of patients [1]; few data exist to guide management in this setting, and available agents, such as anagrelide, may not be as effective as hydroxyurea in reducing the risk of thrombotic and vascular complications in patients with high‐risk ET [3]. As such, there is an unmet need for safe and effective cytoreductive therapy following HU.

In a phase 2 study, the Janus kinase 1/2 inhibitor ruxolitinib was associated with platelet reduction and symptom improvement in patients with high‐risk ET who were intolerant/resistant to hydroxyurea [5]; however, the clinical benefits provided by ruxolitinib in this second‐line setting have not been compared with those provided by hydroxyurea in a first‐line setting. To better understand whether second‐line ruxolitinib in patients with ET intolerant/resistant to hydroxyurea could achieve haematologic profiles comparable with those remaining on first‐line hydroxyurea, we conducted a matched‐cohort analysis comparing clinical outcomes of patients with ET who switched from hydroxyurea to ruxolitinib (HU‐RUX) due to hydroxyurea intolerance/resistance with outcomes of patients who had received hydroxyurea only (HU).

## 2 METHODS

For this post hoc analysis, the HU cohort consisted of patients with ET enrolled in the Myelofibrosis and Essential Thrombocythemia Observational Study (MOST) [6] who received hydroxyurea as their only non‐aspirin ET‐directed therapy for ≥180 days following enrolment. MOST was a prospective observational study that enrolled patients with myelofibrosis or ET at 124 sites throughout the United States from 2016 to 2019 (NCT02953704) [6]. Enrolled patients had a clinical diagnosis of ET per their treating physician, irrespective of meeting the World Health Organization diagnostic criteria, and life expectancy of >6 months. Patients with high‐risk ET (aged ≥60 years and/or a history of TEs) and low‐risk ET (receiving ET‐directed therapy other than aspirin monotherapy at enrolment) were included. Key exclusion criteria included diagnoses of secondary acute myeloid leukaemia, myelodysplastic syndrome, chronic myelogenous leukaemia or secondary thrombocytosis [6]. Patient demographics and physician‐reported baseline data were collected during usual‐care visits over a 36‐month planned observation period, as previously described [6]. The index for the HU cohort was defined as the time of enrolment into MOST.

The HU‐RUX cohort consisted of patients enrolled in a completed phase 2 interventional study of ruxolitinib in patients with ET who were intolerant/resistant to hydroxyurea (NCT00726232) [5]. Eligible patients were aged ≥18 years, diagnosed with ET and either refractory to or had a contraindication to hydroxyurea. Because the European LeukemiaNet criteria for hydroxyurea resistance/intolerance did not exist at the time of initiation of this phase 2 study, reasons for hydroxyurea resistance/intolerance were physician determined and not systematically recorded. Other key inclusion criteria for NCT00726232 included Eastern Cooperative Oncology Group performance status ≤2, platelet count >650 × 10^9^/L unless receiving treatment, absolute neutrophil count ≥1.2 × 10^9^/L and normal renal and hepatic functions. Patients who received treatment with interferon alpha or anagrelide within 7 days, hydroxyurea within 1 day, or other cytoreductive therapies for ET or investigational medications within 28 days of starting ruxolitinib were excluded from the study [5]. The index for the HU‐RUX cohort was defined as the time of initiation of ruxolitinib therapy.

The study protocols and amendments for both studies were approved by the relevant institutional review boards or independent ethics committees. The studies were conducted in accordance with the Declaration of Helsinki, Good Clinical Practice as defined by the International Conference on Harmonisation‒Good Clinical Practice consolidated guidelines, and with applicable regulatory requirements. All patients provided written, informed consent before study participation [5, 6].

Thirty‐nine patients from NCT00726232 were eligible for this analysis and were matched via propensity score (PS) to 627 eligible patients from MOST. Patients from the two studies were matched 1:1 based on age (<60 and ≥60 years), sex, body mass index, ET disease duration, TE history and European Organisation for Research and Treatment of Cancer Quality of Life Questionnaire‐Core 30 (EORTC QLQ‐C30) Global Health Status score at index. These covariates were used to fit a logistic regression model to compute the PS for each patient. Greedy matching was used to find the closest matching patients based on their PS [7]; standardised difference was used to compare the differences in the means between the two groups for each covariate. Descriptive statistics were used to summarise baseline characteristics. Quality of life was measured using the EORTC QLQ‐C30 instrument and data were tabulated with summary statistics. Changes in laboratory values between each study were compared using a *t*‐test and considered significant if *p* < 0.05. Analyses were performed using SAS statistical software, version 9.4 (SAS Institute).

## 3 RESULTS

Of the 39 and 627 patients enrolled in NCT00726232 and MOST, respectively, who were eligible for this analysis, 37 patients from each study were included after PS matching. Two patients from NCT00726232 were excluded from analysis because information was missing for at least one of the matching criteria. Overall, the matched cohorts were well balanced across covariates; the absolute standardised differences for all variables used for matching were within the recommended limit of 0.25 [8], with the exception of EORTC QLQ‐C30, for which the standardised difference was −0.262 (Figure S1 and Table S1). A summary of patient demographics and baseline characteristics is presented in Table 1. The median ages of the two cohorts were comparable (HU‐RUX 50.0 years vs. HU 55.0 years); 27.0% of patients were aged ≥60 years in each cohort. Of patients in the HU‐RUX and HU cohorts, 13.5% and 10.8%, respectively, had a history of TEs before enrolment. The median time from ET diagnosis to index was similar between the two cohorts (HU‐RUX 5.7 years vs. HU 5.3 years). The median (range) follow‐up times for patients in the HU‐RUX and HU cohorts were 93.0 (4.8‒116.6) months and 48.3 (10.3‒61.4) months, respectively. The median (range) ruxolitinib dose for the HU‐RUX cohort was 30.1 (10‒74) mg. Due to the observational nature of MOST, it was not possible to obtain reliable hydroxyurea dosage information from the HU cohort.

**TABLE 1:** Patient demographics and baseline characteristics at enrolment.

Variable	HU‐RUX cohort (*n *= 37)	HU cohort (*n *= 37)	*p*‐Value
Age, median (range) (years)	50.0 (25.0‒87.0)	55.0 (39.0‒87.0)	0.09
≥60, *n* (%)	10 (27.0)	10 (27.0)	1.00
Female, *n* (%)	24 (64.9)	27 (73.0)	0.62
BMI, median (range) (kg/m^2^)	24.3 (17.3‒31.9)	24.5 (17.9‒33.7)	0.88
≥25 kg/m^2^, *n* (%)	16 (43.2)	17 (45.9)	0.94
Time from ET diagnosis to enrolment, median (range) (years)	5.7 (0‒20.9)	5.3 (0.4‒33.5)	0.61
0 to <1 year, *n* (%)	4 (10.8)	2 (5.4)	
1 to <5 years, *n* (%)	12 (32.4)	15 (40.5)	0.55
5 to <10 years *n* (%)	8 (21.6)	11 (29.7)	
≥10 years, *n* (%)	13 (35.1)	9 (24.3)	
History of TE before enrolment, *n* (%)	5 (13.5)	4 (10.8)	1.00
EORTC QLQ‐C30 Global Health Status QoL, *n*	37	37	
Mean (SD)	73.0 (22.9)	78.6 (20.1)	0.26
Patients with leukocyte count (×10^9^/L), *n* ^a^	37	35	
Mean (SD)	9.3 (5.1)	6.8 (3.1)	0.02
Patients with platelet count (×10^9^/L), *n* ^a^	37	35	
Mean (SD)	1027.4 (497.8)	513.9 (154.7)	<0.01
Patients with palpable spleen, *n* ^a^, ^b^	30	26	
Yes, *n* (%)	3 (10.0)	0	
No, *n* (%)	27 (90.0)	25 (96.2)	0.17

Abbreviations: BMI, body mass index; EORTC QLQ‐C30, European Organisation for Research and Treatment of Cancer Quality of Life Questionnaire‐Core 30; ET, essential thrombocythaemia; HU, patients who had received hydroxyurea only; HU‐RUX, patients with hydroxyurea intolerance/resistance who switched to ruxolitinib; QoL, quality of life; SD, standard deviation; TE, thrombotic event.

^a^ Two patients in the HU cohort had missing leukocyte/platelet count data, and 11 and seven patients in the HU and HU‐RUX cohorts, respectively, had missing spleen data at enrolment.

^b^ One patient in the HU cohort reported palpable spleen as indeterminate.

**FIGURE 1:**
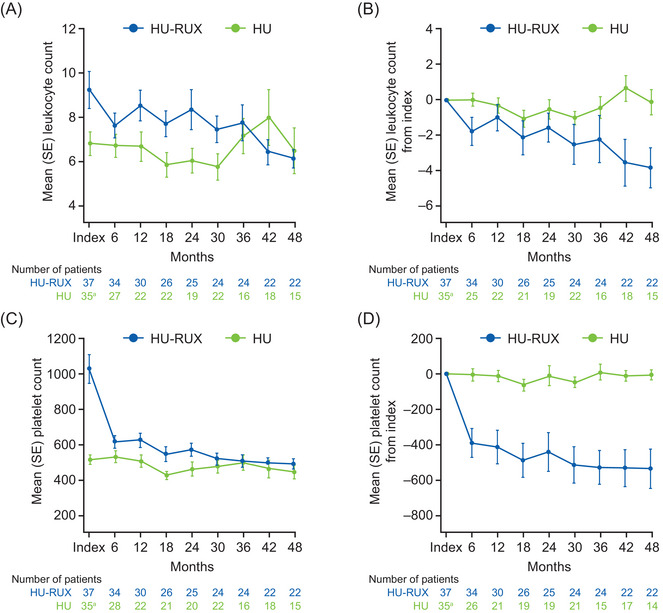
Mean change from index over time in (A and B) leukocyte and (C and D) platelet counts. ^a^Data missing in two patients. HU, patients who had received hydroxyurea only; HU‐RUX, patients with hydroxyurea intolerance/resistance who switched to ruxolitinib; SE, standard error.

At index, mean leukocyte count was higher in the HU‐RUX cohort than in the HU cohort (HU‐RUX vs. HU: 9.3 vs. 6.8 × 10^9^/L; Figure 1A). Figure 1B illustrates relative change in mean leukocyte count from index in each cohort; following index (initiation of ruxolitinib), mean leukocyte count for the HU‐RUX cohort decreased consistently over the entire 48‐month follow‐up period. The mean leukocyte count for the HU cohort remained stable to slightly increased from index throughout the follow‐up period.

The HU‐RUX cohort had higher mean (standard deviation [SD] 0.61) platelet count compared with the HU cohort at index (HU‐RUX vs. HU: 1027 [498] vs. 514 [155] × 10^9^/L; *p *< 0.0001). However, at 6 months post‐index, mean platelet count in the HU‐RUX cohort decreased to levels similar to the HU cohort (HU‐RUX vs. HU: 613.7 [216] vs. 531.1 [178] × 10^9^/L; *p *= 0.1109; Figure 1C) and continued to decline throughout the 48‐month follow‐up period (mean [SD] change from index—6 months: −391.7 [473] × 10^9^/L; 48 months: −539.0 [522] × 10^9^/L; both *p *< 0.001 vs. index; Figure 1D). In the HU cohort, the mean platelet count remained stable from the index date through 48 months (mean [SD] change from index—6 months: −5.7 [175] × 10^9^/L; 48 months: −6.9 [105] × 10^9^/L).

**FIGURE 2:**
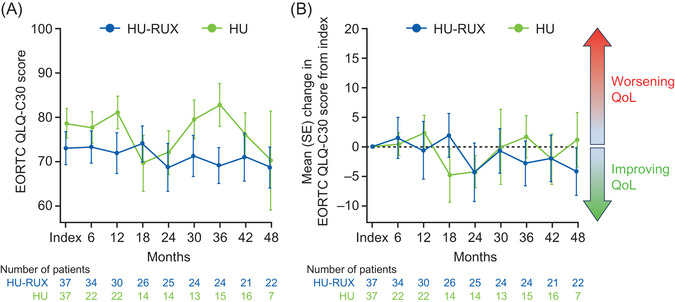
EORTC QLQ‐C30 Global Health Status score throughout the study period (A) and mean change from index (B). EORTC QLQ‐C30, European Organisation for Research and Treatment of Cancer Quality of Life Questionnaire‐Core 30; HU, patients who had received hydroxyurea only; HU‐RUX, patients with hydroxyurea intolerance/resistance who switched to ruxolitinib; QoL, quality of life; SE, standard error.

Of 30 patients in the HU‐RUX cohort with available spleen records, three had palpable splenomegaly at index. The number of patients in the HU‐RUX cohort with palpable splenomegaly decreased to one patient at 12 months and increased to two patients during 18‒36 months, before returning to one patient by 48 months. Of the 26 patients in the HU cohort with spleen records, no patients were reported to have splenomegaly at the index date. Contrary to observations in the HU‐RUX cohort, the number of patients with palpable spleen in the HU cohort increased during the follow‐up period (palpable splenomegaly, *n* = 3). This analysis is limited by the lack of spleen records for >50% of patients in the HU cohort at 24 months. The two cohorts had similar EORTC QLQ‐C30 scores at baseline that did not change considerably over time (Figure 2).

During the study period, three patients (8.1%) in the HU‐RUX cohort experienced a vascular complication and one patient (2.7%) experienced two haemorrhagic events, one of which was grade ≥3. Of the three patients with vascular complications, two had arterial events (transient ischaemic attack and cerebrovascular accident, *n* = 1 each) and one had a venous event (pulmonary embolism), which occurred at 39.4, 39.2 and 35.8 months from index, respectively; at the last assessment before the vascular complication in these three patients, leukocyte counts were 3.6 × 10^9^, 5.3 × 10^9^ and 5.5 × 10^9^/L, respectively; platelet counts were 519 × 10^9^, 593 × 10^9^ and 516 × 10^9^/L, respectively. The patient who experienced a cerebrovascular accident had a history of thrombophlebitis. This patient did not receive aspirin at index; the two other patients with vascular complications had no history of TEs before enrolment and were receiving aspirin at index. No thrombotic or haemorrhagic events were reported in the HU cohort. No deaths related to TEs or haemorrhagic events were reported in either cohort throughout follow‐up.

## 4 DISCUSSION

The optimal cytoreductive therapy for patients with ET who are refractory or intolerant to hydroxyurea remains an unmet clinical need; the present results support that patients who receive ruxolitinib as second‐line treatment following hydroxyurea due to resistance/intolerance achieved similar haematological responses as those attained by patients receiving first‐line hydroxyurea; despite higher mean platelet counts of the HU‐RUX cohort at index, mean platelet count became comparable 6 months after initiation of ruxolitinib. Moreover, splenomegaly reduced in the HU‐RUX cohort but increased in the HU cohort over the observation period. Three patients (8%) in the HU‐RUX cohort experienced TEs, whereas no TEs were reported in the HU cohort. The observation that ruxolitinib provided durable platelet and leukocyte reductions in this second‐line setting is consistent with the results of the phase 2 MAJIC‐ET study that compared best‐available therapy versus ruxolitinib in patients with ET who were resistant/intolerant to hydroxyurea, in which 93% of patients in the ruxolitinib arm achieved objective haematologic response (46.5% each achieved complete and partial responses), whereas symptom improvement was greater in patients who received ruxolitinib versus best‐available therapy [9]. Together, these findings provide the foundation for future studies designed to further investigate the role of ruxolitinib as a second‐line treatment for ET in the setting of hydroxyurea intolerance or resistance.

Results of this study should be interpreted in context of its limitations; despite our effort to control for the differences of the two cohorts by closely matching the baseline characteristics, the two cohorts were derived from separate studies with distinct eligibility criteria. Moreover, differences in blood counts and splenomegaly at index were potentially impacted by the fact that patients were required to have discontinued ET‐directed cytoreductive therapy within 28 days of enrolment in NCT00726232 (HU‐RUX cohort) [5], whereas this was not required for enrolment in MOST (HU cohort). Furthermore, PS matching cannot account for possible biases due to differences in clinical management of patients in a controlled clinical trial (HU‐RUX) compared with a prospective observational study (HU). For example, clinical assessments such as blood counts and physical examinations in the HU‐RUX cohort took place at pre‐specified time points [5], whereas assessments in patients from the HU cohort occurred per usual care [6]. In addition, this study was not adequately powered to detect differences in TE incidence between the two cohorts due to small sample size.

In conclusion, the results from this matched‐cohort study suggest that patients who need to switch from hydroxyurea owing to intolerance or resistance may derive benefit from ruxolitinib treatment that is comparable with those remaining on hydroxyurea. Future studies comparing the clinical outcomes of patients with ET who switched from hydroxyurea to ruxolitinib or to second‐line therapy such as anagrelide or interferon, in the setting of hydroxyurea resistance or intolerance would provide further insights.

## AUTHOR CONTRIBUTIONS

Michael R. Grunwald, Ellen K. Ritchie, Elisa Rumi and Francesco Passamonti participated in the collection, interpretation and analysis of the data and the development of the manuscript draft. Albert Assad, J. E. Hamer‐Maansson, Jingbo Yu, Tricia Kalafut and Evan Braunstein participated in the study design and conduct, the interpretation and analysis of the data and the development of the manuscript draft. All authors approved the final manuscript for submission.

## CONFLICT OF INTEREST STATEMENT

Michael R. Grunwald has received consulting fees from AbbVie, Agios/Servier, Amgen, Astellas Pharma, Blueprint Medicines, Bristol Myers Squibb, Cardinal Health, Daiichi Sankyo, Gamida Cell, Genentech, Gilead Sciences, GlaxoSmithKline/Sierra Oncology, Incyte Corporation, Invitae Corporation, Jazz Pharmaceuticals, Novartis, Ono Pharmaceutical, Pfizer, Pharmacosmos, Premier, Sobi/CTI BioPharma and Stemline Therapeutics; has received research support from Incyte Corporation and Janssen; and has stock ownership in Medtronic. Ellen K. Ritchie has served as a consultant for Celgene, Incyte Corporation and Novartis; is on the speaker's bureau for ARIAD Pharmaceuticals, Celgene, Incyte Corporation and Novartis; has received research funding from Astellas Pharma, Bristol Myers Squibb, Novartis, NS Pharma and Pfizer; and has received travel support from Celgene and Novartis. Elisa Rumi has served as a consultant for Novartis. Albert Assad, J. E. Hamer‐Maansson, Jingbo Yu, Tricia Kalafut and Evan Braunstein are employees of and stockholders in Incyte Corporation. Francesco Passamonti has received honoraria from AbbVie, AOP Health, Bristol Myers Squibb, Celgene, Janssen, Karyopharm Therapeutics, Kyowa Kirin, MEI Pharma, Novartis and Roche; has served on the advisory board of AbbVie, AOP Health, Bristol Myers Squibb, Celgene, Janssen, Karyopharm Therapeutics, Kyowa Kirin, MEI Pharma, Novartis and Roche; and has received research funding from Bristol Myers Squibb.

## ETHICS STATEMENT

The protocols and all amendments for NCT00726232 and MOST (NCT02953704) were reviewed and approved by qualified institutional review boards/independent ethics committees before enrolment of participants in the study.

## PATIENT CONSENT STATEMENT

All patients provided informed written consent prior to study enrolment.

## PERMISSION TO REPRODUCE MATERIAL FROM OTHER SOURCES

The authors own copyright of the figures included in this manuscript; no permissions to reproduce are required..

## CLINICAL TRIAL REGISTRATION

ClinicalTrials.gov: NCT02953704; NCT00726232.

## Supporting information

Supporting Information

## Data Availability

Incyte Corporation (Wilmington, DE, USA) is committed to data sharing that advances science and medicine while protecting patient privacy. Qualified external scientific researchers may request anonymised datasets owned by Incyte for the purpose of conducting legitimate scientific research. Information on Incyte's clinical trial data sharing policy and instructions for submitting clinical trial data requests are available at: https://www.incyte.com/Portals/0/Assets/Compliance%20and%20Transparency/clinical-trial-data-sharing.pdf?ver%20=%202020-05-21-132838-960.
