# Supplementary material for: Treatment comparison of hydroxyurea versus ruxolitinib in essential thrombocythaemia: A matched‐cohort analysis
Source: EJHaem. 2024 Jul 19;5(4):778–83. doi: 10.1002/jha2.954 (PMC11327719; doi:10.1002/jha2.954)
Supplement: Supplementary file 1 — Supporting Information [file JHA2-5-778-s001.docx]

**SUPPORTING INFORMATION (GRUNWALD ET AL)**

**FIGURE S1** Covariate balance of the covariables included in the propensity score model. Abbreviations: BMI, body mass index; EORTC QLQ-C30, European Organisation for Research and Treatment of Cancer Quality of Life Questionnaire-Core 30; HU, patients who had received hydroxyurea only; HU-RUX, patients with hydroxyurea intolerance/resistance who switched to ruxolitinib; Obs, observation; TE, thrombotic event.


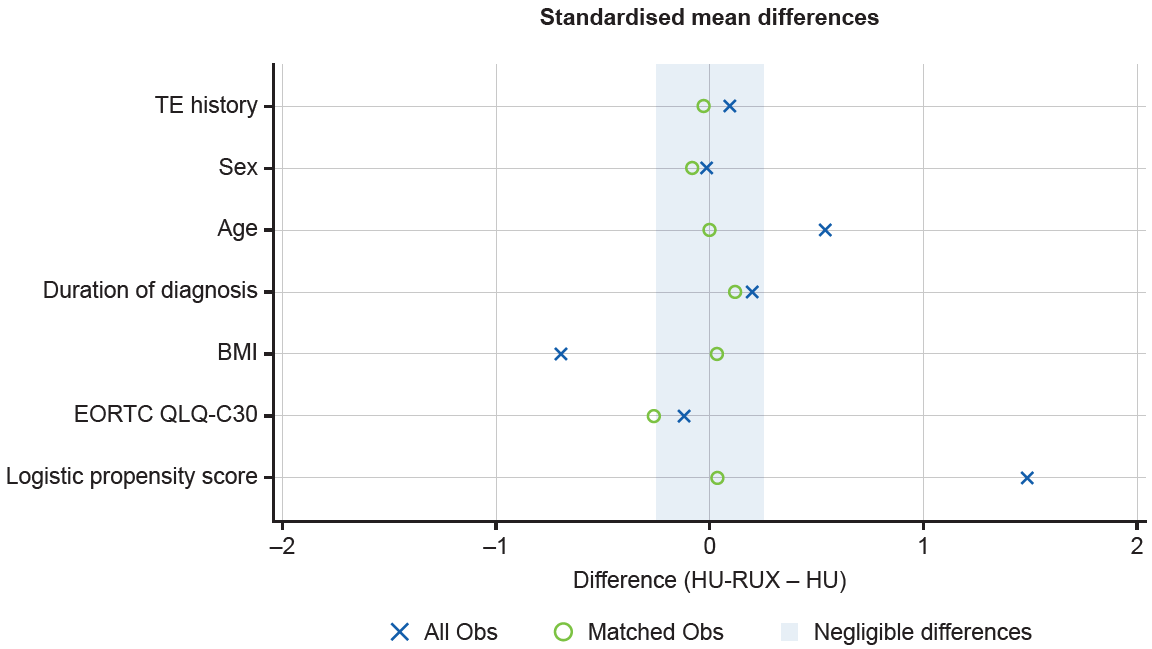


**TABLE S1** Standardised mean differences (HU-RUX − HU) of matching parameters before and after propensity score matching.

| **Matching parameters** | **Standardised mean difference**  **before PS matching** | **Standardised mean difference**  **after PS matching** |
| --- | --- | --- |
| Overall | 1.487 | 0.036 |
| EORTC QLQ-C30 score at baseline | −0.117 | −0.262 |
| Time from diagnosis to enrolment | 0.199 | −0.120 |
| BMI | −0.694 | 0.035 |
| Age group | 0.542 | 0 |
| Sex | −0.014 | −0.081 |
| TE history | −0.095 | −0.027 |

Abbreviations: BMI, body mass index; EORTC QLQ-C30, European Organisation for Research and Treatment of Cancer Quality of Life Questionnaire-Core 30; HU, patients who had received hydroxyurea only; HU-RUX, patients with hydroxyurea intolerance/resistance who switched to ruxolitinib; TE, thrombotic event.
